# Pre‐stimulus activities affect subsequent visual processing: Empirical evidence and potential neural mechanisms

**DOI:** 10.1002/brb3.3654

**Published:** 2025-02-05

**Authors:** Narjes Soltani Dehaghani, Mojtaba Zarei

**Affiliations:** ^1^ Institute of Medical Science and Technology Shahid Beheshti University Tehran Iran; ^2^ Department of Neurology Odense University Hospital Odense Denmark; ^3^ Department of Clinical Research University of Southern Denmark Odense Denmark

**Keywords:** arousal, brain oscillations, cognitive functions, feature‐based attention, mental imagery, pre‐stimulus activities, spatial attention, temporal attention

## Abstract

**Purpose:**

Humans obtain most of their information from visual stimuli. The perception of these stimuli may be modulated by the ongoing pre‐stimulus brain activities. Depending on the task design, the processing of different cognitive functions such as spatial attention, feature‐based attention, temporal attention, arousal, and mental imagery may start prior to the stimulus onset.

**Method:**

This process is typically accompanied by changes in pre‐stimulus oscillatory activities including power, phase, or connectivity in different frequency bands. To explain the effect of these changes, several mechanisms have been proposed. In this article, we review these changes and the potential mechanisms in the context of the pre‐stimulus enabled cognitive functions. We provide evidence both in favor of and against the most documented mechanisms and conclude that no single mechanism can solely delineate the effects of pre‐stimulus brain activities on later processing. Instead, multiple mechanisms may work in tandem to guide pre‐stimulus brain activities.

**Finding:**

Additionally, our findings indicate that in many studies a combination of these cognitive functions begins prior to stimulus onset.

**Conclusion:**

Thus, dissociating these cognitive functions is challenging based on the current literature, and the need for precise task designs in later studies to differentiate between them is crucial.

## Graphical abstract

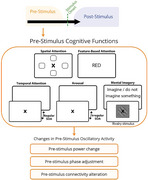

## 1 INTRODUCTION

The brain never rests. Classically, brain activities are grouped into ongoing activity versus stimulus‐induced activity. Beyond this dichotomy, some studies have investigated the brain activities prior to the stimulus onset, referred to as pre‐stimulus brain activity. Historically, pre‐stimulus brain activity was considered mere noise (Sherrington, 1906). However, within the last two decades, a consensus has been reached that pre‐stimulus activity can significantly impact the processing of an incoming stimulus and behavioral performance (Başar et al., 1998; Busch et al., 2009; Fries et al., 2001).

While there is a plethora of studies that have shown the effect of pre‐stimulus brain activities on various post‐stimulus processing, the cognitive functions that are activated prior to stimulus onset are rarely explicitly discussed. This review focuses on the most probable cognitive functions that are enabled in the pre‐stimulus interval. Specifically, we concentrated on pre‐stimulus selective spatial and feature‐based attention, temporal attention, arousal, and mental imagery. We describe common task designs, changes in pre‐stimulus oscillatory activities, how behavior is modulated by these changes, and the suggested mechanisms by previous studies (if any) that explain the impacts of these changes on further processing in the context of any of the mentioned cognitive functions.

**FIGURE 1:**
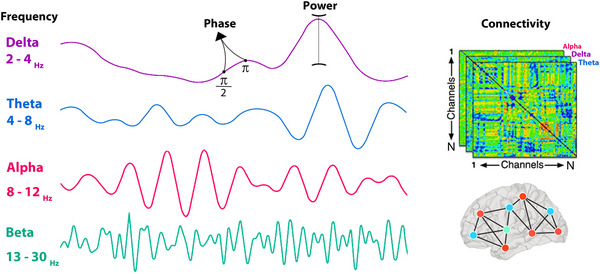
The concepts of frequency, phase, power (left), and connectivity between channels or brain regions (right).

In the current review, we will mainly focus on the studies that used Electroencephalography (EEG) or Magnetoencephalography (MEG) recording to study pre‐stimulus oscillations in relation to the enabled cognitive function. The term “oscillations” refers to rhythmic fluctuations in the excitability of populations of neurons, which are described by frequency, power, and phase information (M. X. Cohen, 2014). Frequency is the speed of the oscillation, power is the amount of energy in a frequency band, and phase reflects the timing of activity within a neural population and is expressed as a point in an oscillatory period between 0 and 2π. Brain oscillations are traditionally grouped into frequency bands including delta (2–4 Hz), theta (4–8 Hz), alpha (8–12 Hz), beta (13–30 Hz), and gamma (30–100 Hz). In the current review, we will describe different kinds of changes that can happen in pre‐stimulus brain activities including changes in power and phase of different frequency bands as well as connectivity patterns between certain brain regions in the time‐frequency domain prior to stimulus onset. Among different frequency bands, alpha is the most reported oscillation prior to stimulus onset; however, activation of pre‐stimulus delta, theta, and beta has also been reported by previous studies. Figure 1 represents the concepts of frequency, power, phase, and connectivity.

One of the main mechanisms that describes the effect of different kinds of changes in pre‐stimulus brain activities on later processing is called the gating mechanism, also known as gain control mechanism. For instance, the impact of changes in pre‐stimulus power and phase of brain oscillations in electrophysiological studies as well as changes in pre‐stimulus BOLD responses during fMRI studies can be explained by the gating mechanism. In this context, change in the power of pre‐stimulus oscillations is mainly seen in the range of the alpha frequency band. The traditional idea is that alpha power is decreased in sensory brain regions responsible for the processing of an incoming stimulus, while concurrently increased in irrelevant sensory brain regions to inhibit distracting information (so‐called *inhibitory gating mechanism*). Additionally, the effect of changes in the pre‐stimulus phase of oscillatory activity, for example, while covert temporal sampling, can be explained by the gating mechanism. Time is a fundamental dimension across various cognitive functions that could be implemented through the temporal sampling of the environment. Sampling provides an optimized yet abstract format for information representation and storage. The temporal sampling of the external stimulation by our sensory organs occurs overtly (e.g., saccadic eye movements) or covertly. The pre‐stimulus gating mechanism, covert temporal sampling can happen while pre‐stimulus phase adjustment of brain fluctuations. More specifically, the moments of heightened excitability, which corresponds to a particular oscillatory phase, become aligned with the timing of an incoming expected event (A. C. Nobre & Van Ede, 2018; Schroeder & Lakatos, 2009). Based on this notion, a new mechanism called perceptual cycles was introduced according to which human perception may operate in successive periodic cycles alternating between phases of optimal excitability, usually followed by improvement in behavioral performance, and phases associated with stronger suppression at which typically a decline of behavioral performance is noticed (VanRullen, 2016). In the case of fMRI studies, the gating mechanism is usually used to describe the effects of increased pre‐stimulus Blood oxygen level dependent (BOLD) signal in the relevant brain regions responsible for processing an incoming stimulus and concurrent decrease of pre‐stimulus BOLD response in unrelated brain regions. Another common mechanism that is used to describe the effects of pre‐stimulus brain activities on later processing is called sequential spatial sampling, which is mainly reported in spatial attentional paradigms (Landau et al., 2015). In this article, we will review these three widely reported mechanisms regarding the enabled pre‐stimulus cognitive function. We believe that although each of these mechanisms can partially explain the impact of pre‐stimulus brain activities on further processing, none of these mentioned mechanisms nor any other presented mechanism can solely explain all the observed effects. In the same vein, it is becoming increasingly clear that multiple mechanisms may work simultaneously to guide pre‐stimulus brain activities (A. C. Nobre & van Ede, 2023; Jensen, 2023).

Pre‐stimulus activities may affect further processing in any sensory modality, and these effects are similar in principle across different modalities (Frey et al., 2016; Hong et al., 2014; McGinley et al., 2015; Waschke et al., 2019; Weisz et al., 2014). However, in this article, we will mainly focus on visual information processing and consider the pre‐stimulus period up to a few seconds prior to the stimulus presentation. A longer pre‐stimulus period is often relevant to long‐term memory and contextual learning, using multiple experiments in a single day or over longer periods (Chun & Jiang, 1998; André M Cravo et al., 2017; J. J. Summerfield et al., 2006; Mattiesing et al., 2017), which is not included in the current article. In addition, working memory, although it may be enabled prior to stimulus onset (Bae & Luck, 2018; Foster et al., 2017, 2016), is not included in this article as there are already some excellent reviews on this subject (Addante et al., 2015; N. Cohen et al., 2015; van Ede et al., 2017).

## 2 PREPARATORY ATTENTION

Preparatory attention is probably the most common cognitive phenomenon that is activated before stimulus onset. Preparatory attention refers to the selective attentional activities before the presentation of a stimulus and is classically separated into spatial versus feature‐based (also called content‐based) attention (Battistoni et al., 2017). In pre‐stimulus spatial attention, attention is diverted to a specific point in space, while in pre‐stimulus feature‐based attention, attention is directed to a particular feature of the incoming stimulus, although the location of an upcoming stimulus can also be regarded as a feature. A frequently used task design for preparatory attention is the use of a cue before stimulus presentation. The cue could be valid, that is, giving correct information about the incoming stimulus, invalid (misleading), or neutral. The consensus is that presenting a valid cue before stimulus onset improves behavioral performance; however, at least one report indicated that valid cues may hinder performance depending on the specific low‐level features of the incoming stimulus (Yeshurun & Carrasco, 1998).

### 2.1 Pre‐stimulus spatial attention

#### 2.1.1 Task design

In the case of spatial attention, the cue may appear at the location where the impending target or distractor is presented using variations of Posner cueing paradigms (Posner, 1980). Alternatively, it could be presented at the center but guiding (distracting) the participants to the location of the incoming stimulus, for example, using an arrow, which points to a certain location in the visual field. The cue may appear at any location in the visual field (Noonan et al., 2016; Ruff & Driver, 2006). Another common task design is when participants are cued to alternatively shift their attention to the left or right of a central fixation point in each trial, while the stimulus can be presented at both attended and unattended locations (Milton & Pleydell‐Pearce, 2016; Thut et al., 2006). Such paradigms allow examining how pre‐stimulus fluctuations vary in task‐relevant (contralateral hemisphere) versus task‐irrelevant (ipsilateral hemisphere) brain regions and their possible influences on further processing.

Spatial attention is divided into covert versus overt spatial attention. In overt spatial attention, participants can move their eyes toward the presented cue or stimulus, whereas in covert spatial attention, little or no eye movement occurs (Lowet et al., 2018). Thus, eye tracking is crucial to distinguish between these two types of attention, while participants perform these tasks.

Overt spatial attention is mainly studied during pre‐saccadic attention (not to be confused with pre‐stimulus) (Li, Hanning, et al., 2021; Li, Pan, et al., 2021). In pre‐saccadic paradigms, the stimulus occurs between the cue and the saccade (Li et al., 2016; Rolfs & Carrasco, 2012), making it different from pre‐stimulus brain activities. Research about pre‐stimulus overt spatial attention is typically confined to a few studies, which assessed overt spatial attention by displaying a cue before the stimulus (Griffiths & Le Pelley, 2019; Kuhn & Benson, 2007). In this context, some authors presented deviated eyes in the central visual field to guide participants' saccade. In such a paradigm, the participants may look in the direction of the deviated eyes, even when the cue is invalid (Kuhn & Kingstone, 2009). Both bottom‐up (stimulus‐driven) and top‐down (goal‐driven) mechanisms may be recruited to steer pre‐stimulus overt spatial attention in these task designs (Kaspar, 2013). In a recent study, participants were cued to overtly attend to the left or right of the incoming stream, an increase of pre‐stimulus alpha power was predictive of a conscious perception of the impending event (Menétrey et al., 2023). However, attributing the results of this study solely to pre‐stimulus overt attention may not be accurate as pre‐stimulus temporal and feature‐based attention were also enabled in their experiment.

**FIGURE 2:**
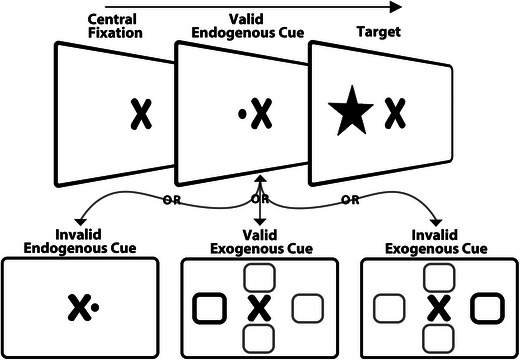
Exemplar task design for covert spatial attention.

Contrary to overt spatial attention, evidence of pre‐stimulus covert spatial attention is abundant (Barbot et al., 2012; Herrmann et al., 2010; Yeshurun & Rashal, 2010). Covert spatial attention is divided into endogenous (sustained and voluntary) and exogenous (transient and involuntary) attention (Carrasco, 2018). Classically, endogenous attention is known as a top‐down (goal‐driven) process and exogenous attention as a bottom‐up (stimulus‐driven) process (Bowling et al., 2020; Corbetta & Shulman, 2002; Nakayama & Mackeben, 1989). Exogenous attention depends on factors external to the participants, for example, a salient peripheral cue, and is associated with sensory stimulation. In comparison, endogenous attention depends on individuals' expectations and intentions and is focused, for example, by using a centrally presented directional cue (Giordano et al., 2009; Jigo & Carrasco, 2020; Klein et al., 1992). Figure 2 shows a typical pre‐stimulus spatial attention paradigm.

#### 2.1.2 Mechanisms

It is important to note that while some studies observed the dichotomy of exogenous versus endogenous in their task design, many pre‐stimulus studies did not consider this dichotomy because usually a combination of bottom‐up and top‐down factors is entailed in spatial covert attentional paradigms. Thus, in further explanations of this section, we have ignored this dichotomy.

Before reviewing the mechanisms of how pre‐stimulus brain activities affect further processing during the activation of pre‐stimulus covert spatial attention, it is worth noting that in many experiments, pre‐stimulus covert spatial attention and feature‐based attention are concurrently in play. For example, many tasks manipulated the location of an incoming stimulus and dissociated the target from distractor stimuli. This means that both spatial and feature‐based attention were active in these experiments, because the differentiation between the target and the distractor depended on some features, in addition to the spatial feature. Another example is when participants are instructed to look for a specified feature that may appear in different locations. What makes it even more complicated is when pre‐stimulus temporal attention is also involved in the process (see Section 3).

Most of the studies indicated that pre‐stimulus covert spatial attention facilitates sensory processing in the brain via a causally sensory inhibitory gating mechanism. In the context of the inhibitory gating mechanism, general findings based on Functional Magnetic Resonance Imaging (fMRI) studies show increased pre‐stimulus brain activity contralateral to the cued location in comparison to ipsilateral sensory brain regions (Battistoni et al., 2017). Considering the electrophysiological studies, in many studies participants were cued to alternatively shift their attention between the left or right of the visual field prior to the presentation of target and/or distractor stimuli. The general finding of these studies was that pre‐stimulus alpha power increased in the ipsilateral occipital cortex to inhibit distractors, that is, filtering competing sensory inputs prior to stimulus onset, and concurrently alpha power decreased in the occipital cortex contralateral to the attended location (Jensen & Mazaheri, 2010). This effect of pre‐stimulus alpha rhythms was found to be independent of cue modality (Frey et al., 2015) and is typically followed by an improvement of behavioral performance. Thus, many studies concluded that pre‐stimulus changes in alpha power implement attentional control, that is, a causal relationship between pre‐stimulus alpha power and attention.

More recent studies reported that pre‐stimulus covert spatial attention was not always implemented via the sensory inhibitory gating mechanism. In this context, it was suggested that shifting attention to the left or right in different trials would make it hard to separate the inhibitory and facilitatory effects of pre‐stimulus activities. They also suggested that if only two locations were considered as the locations where the target stimulus could appear, the experimenter should avoid informing the participants about the presence of a competing distractor in the visual field contralateral to the target stimulus in a single trial, because this paradigm would prevent discriminating the possible facilitatory effect of the attention from distractor inhibition, as it might sharpen the focus of participants' attention to the target (Noonan et al., 2016). To separate the effects of target facilitation from distractor suppression, another study used a task design in which only one visual hemifield was involved in all trials and the infrequent target stimuli always appeared in the attended location (Slagter et al., 2016). These authors did not find any pre‐stimulus inhibitory fluctuations and thus suggested that the inhibitory role of pre‐stimulus alpha oscillations might only be present in competitive task designs. This study contrasts with the previous one (Noonan et al., 2016), in which the location of distractors was changed between trials or was constant across the block, leading to the distractor inhibition effect only in the block design. We suggest that the main reason behind this contradiction is the presence of distractors in the latter study and no distractor (only rare targets) in the former one.

Some authors suggested that a key limitation of the studies that used spatial attention paradigms is that they considered alpha power without manipulating the attended locations and their timing in the presence of distractor versus no distractor conditions, while alpha‐band activity is critical in tracking location and timing of spatial covert attention (Foster & Awh, 2019). They suggested that target facilitation and suppression of task‐irrelevant information may not simply be different sides of the same coin, meaning that pre‐stimulus alpha changes may only account for target enhancement rather than distractor suppression. In support of this notion, a recent study used an edited version of the frequency tagging paradigm in the early visual cortex and concurrently examined posterior alpha modulation, while the subjects performed a distractor suppression task (Ferrante et al., 2023). They reported no evidence of attentional distractor suppression associated with pre‐stimulus alpha power increase and stated that there might be another gating mechanism that is not related to attention per se. Another study suggested that the increase of alpha power in irrelevant brain regions is at least partially explained by a secondary complementary mechanism driven by the load of experiment‐relevant information (Jensen, 2023). Although the findings of the latter study were tested in the post‐stimulus interval, its findings are compatible with previous reports in pre‐stimulus attentional paradigms. In this context, providing high uncertainty about the location of targets and distractors, for example, by specifying more than two possible locations in which the target and distractor may appear, could further help to dissociate target versus distractor processing (Noonan et al., 2016).

Additionally, recent studies have started to cast doubt on the causal relationship between pre‐stimulus alpha power and attention. In this context, it is stated that if changes in alpha power are to implement attentional control, they should also explain the variance in the degree of post‐stimulus attentional‐related modulation of neural responses (Morrow et al., 2023). So far, only a few studies have tested this phenomenon, mainly by looking at the correlation between pre‐stimulus alpha power change and post‐stimulus attentional evoked response. The findings of these studies are contradictory. Some authors could not find this relationship and thus denied any causal effect of pre‐stimulus alpha power on attention (Antonov et al., 2020; Gundlach et al., 2020). They suggested that pre‐stimulus alpha changes could be a product of selective attention, or they may represent an epiphenomenon of a latent process or other aspects of attention such as noise reduction. In contrast, some other authors provided evidence in favor of a relationship between pre‐stimulus alpha power and sensory/perceptual attentional evoked responses (Gould et al., 2011; Popov et al., 2017) or post‐perceptual processes (Grent‐’t‐Jong et al., 2011; Zhao et al., 2019), thus supporting the causal effect of pre‐stimulus alpha power on attention. A recent review assessed the above controversy and concluded that the causal role of pre‐stimulus alpha power changes on covert spatial attention could not be determined due to a limited number of studies (Morrow et al., 2023). They suggested using Transcranial Magnetic Stimulation (TMS) or similar methods to scrutinize a potential causal relationship. We suggest additional difficulties that stem from the interaction between arousal, spatial, feature‐based, and temporal attention in many task designs.

Apart from sensory regions, pre‐stimulus attentional facilitation can also take place in motor cortices. According to the premotor theory of attention (Rizzolatti et al., 1987; Smith & Schenk, 2012), any shift of overt and covert spatial attention is preceded by motor activation. Although more recent studies indicate that visual spatial attention is not limited to the oculomotor system (Hanning et al., 2019), evidence in favor of tight coupling between visual selective attention and the oculomotor system is abundant (Fiebelkorn & Kastner, 2019; Heide & Kömpf, 1998; Posner et al., 1982). In this context, the dorsal frontoparietal network is involved in oculomotor control, particularly in the beta frequency band (Gunduz et al., 2011; Schubotz, 2007). Similarly, multiple M/EEG studies have reported beta power changes prior to stimulus onset (de Vries et al., 2019; Sokoliuk et al., 2019) but assigning these changes solely to covert spatial attention is not possible due to multiple confounding cognitive functions such as temporal attention and working memory. However, to our knowledge, at least one Electrocorticography (ECoG) study has reported a direct association between the reduction of pre‐stimulus beta power in the motor cortex and subsequent covert attention to a relevant location (Gunduz et al., 2011).

The phase of pre‐stimulus oscillations can also change during activation of pre‐stimulus covert spatial attention. In a study, (Busch et al., 2009) demonstrated how a cue prior to the presentation of a visual near‐threshold (NT) stimulus, which could be displayed on the right or left side of a fixation point in each trial, influenced perceptions. The target was presented in 80% of the trials. The results indicated that the phase of pre‐stimulus alpha oscillation, mainly at 8 Hz, predicted whether participants perceived the upcoming stimulus or not. In another study, two gratings were presented in the two visual hemifields, and participants had to detect a contrast decrement in the gratings during maintaining fixation on a central dot (Landau et al., 2015). Their results indicated that the pre‐stimulus theta phase, mainly at 4 Hz, was predictive of detected versus missed incoming targets. The findings of these studies reveal that spatial attention may sequentially sample the visual environment. This means that the 8 Hz sampling mechanism—detected when a single location was relevant in each trial—is divided by two when two locations were relevant in each trial. In other words, when participants are required to attend to only one stimulus in each trial, this stimulus is sampled eight times per second, and when two locations are to be attended in each trial, each stimulus is sampled four times per second.

More recently a study examined pre‐stimulus phasic modulation for unattended versus attended locations by presenting spatially cued (attended) and uncued (unattended) targets to the participants (Harris et al., 2018). Their results indicated that the pre‐stimulus phase of theta oscillations modulated the detection of unattended more than attended locations, reflecting that pre‐stimulus covert spatial attention performed sampling via theta phase. In comparison, they did not find any attention‐related difference in the alpha phase for attended versus unattended stimuli, thus proposing that the pre‐stimulus alpha phase represents a perceptual rather than attentional role. We suggest that although in this study participants were cued to covertly attend to a single location, the fact that the stimulus could be presented at both attended and unattended locations might have the same effect as sequential spatial sampling and thus the observed effect in theta phase could at least partially be attributed to the division of alpha sampling to two locations.

Moreover, pre‐stimulus attentional oscillations may also contribute to large‐scale synchronization, that is, distant communication between brain regions. Using a Posner cueing task, pre‐stimulus high alpha phase synchronization, which started shortly after cue onset, was shown to coordinate neural processing across frontoparietal and visual systems during activation of pre‐stimulus endogenous covert spatial attention (Lobier et al., 2018). This synchronization co‐varied with neural and behavioral evaluations of attention, and the authors noted that the observed connectivity pattern may underlie anticipatory endogenous control of attention through facilitating communication between relevant cortical areas. In another study, subjects performed a multi‐object tracking task while keeping a fixation on the center of mass and searched for a feature change (Rouhinen et al., 2020). The results showed that the strength of pre‐stimulus phase connectivity between the visual, posterior parietal, and prefrontal cortices was predictive of attentional capacity in theta, alpha, and gamma‐band frequencies, thereby enabling effective neural communication. However, assigning the found effects to pre‐stimulus covert spatial attention alone may not be true as pre‐stimulus feature‐based and sustained attention were also enabled prior to stimulus onset in this study.

**FIGURE 3:**
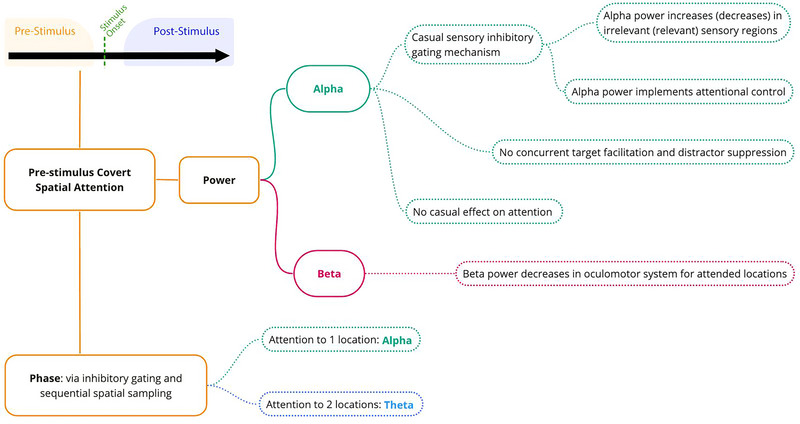
Changes in power and phase of different frequency bands associated with the activation of pre‐stimulus covert spatial attention.

Regarding alpha power, phase, and inter‐regional synchrony are frequently mentioned in the context of pre‐stimulus target‐cueing paradigms in covert spatial attention. Peylo et al. (2021) proposed a model in which inter‐regional alpha phase synchrony in the frontoparietal network controls the alpha power in the parieto‐occipital cortex. If alpha power in the parieto‐occipital cortex is increased (decreased), it will subsequently prolong (shorten) the inhibitory phase of pre‐stimulus oscillations, and this leads to deterioration (improvement) of behavioral responses. Although this model has integrated the results from various characteristics of alpha oscillations, it is based solely on alpha oscillations and does not provide explanations about distractor‐cueing paradigms. Figure 3 summarizes the changes in pre‐stimulus oscillatory activity associated with the activation of covert spatial attention prior to stimulus onset.

### 2.2 Pre‐stimulus feature‐based attention

Considering preparatory feature‐based attention, a valid cue before stimulus presentation informs the participant about a feature of the impending stimulus, while an invalid cue does not align with the participant's expectations concerning the stimulus feature. Feature‐based attention is typically described as a top‐down, endogenous mechanism (Donovan et al., 2020); however, since feature‐based and spatial attention are often intertwined, bottom‐up feature‐based attention may also play a role (Jia et al., 2022). Depending on the type of feature, preparatory feature‐based attention can engage at various levels of stimulus content, ranging from simple features to objects, or even superordinate categories.

In principle, brain activities in pre‐stimulus feature‐based attention are similar to those in pre‐stimulus spatial attention. For instance, pre‐stimulus BOLD activity increases in regions that selectively respond to attended characteristics of the stimulus during the activation of feature‐based attention before stimulus presentation (Battistoni et al., 2017). For example, when pre‐stimulus cues informed participants about an imminent upcoming image of a face or a house, pre‐stimulus BOLD activity increased in stimulus‐related cortical brain regions (fusiform area for faces and para‐hippocampal area for houses) (Puri et al., 2009). Some studies interpret this effect as “sensory templates”, meaning that preparatory attention evokes brain activities that resemble those during the perception of the same stimulus (Gayet & Peelen, 2022; Stokes et al., 2009). However, other studies have shown that while some facilitatory effects are observed during the pre‐stimulus time interval, they cannot be attributed to sensory templates, as they do not resemble post‐stimulus evoked responses. Instead, they reflect a “non‐sensory template”, suggesting an association with an abstraction of the attended feature (Gong et al., 2022). The concept of sensory templates in pre‐stimulus feature‐based attention has also been examined in MEG studies; some reported the presence of pre‐stimulus sensory templates (Kok et al., 2017), while others denied their existence (Meijs et al., 2019).

Potential explanations for the divergence between sensory and non‐sensory templates include using familiar versus unfamiliar objects in these studies, as well as general differences in task designs. For example, finding the sensory template for an impending stimulus in the pre‐stimulus time interval of the same trial in a single task (Gayet & Peelen, 2022) versus finding no such sensory template when searching for the template retrieved from a baseline task in the pre‐stimulus period of another feature‐based attention task (Gong et al., 2022). The latter case may be attributed to the different effects of expectations versus attention (Rungratsameetaweemana & Serences, 2019).

Electrophysiological studies of pre‐stimulus feature‐based attention have shown that alpha band power is decreased in the brain regions involved in the processing of the attended feature and is increased over the irrelevant brain regions in the pre‐stimulus time interval, reflecting an inhibitory gating mechanism (Frey et al., 2015). However, to our best knowledge, no study has specifically addressed whether various changes in alpha power in pre‐stimulus feature‐based attention are directly linked, are independent, or if alpha power changes prior to stimulus onset have a causal role in pre‐stimulus feature‐based attention. Although many studies focused generally on preparatory or selective attention, they primarily investigated or discussed effects attributed to pre‐stimulus spatial attention, although mixed effects of feature‐based attention were also usually present (Antonov et al., 2020; Morrow et al., 2023). The main findings of these studies were reported in the pre‐stimulus covert spatial attention section of the current paper; thus, we avoid repeating them here.

We could not find any evidence of a relationship between pre‐stimulus feature‐based attention and beta power changes in motor cortices prior to stimulus onset. However, a recent study found a relationship between pre‐stimulus ocular preparation and subsequent feature‐based attention, without testing the possible involvement of beta oscillations (Wen et al., 2021). Additionally, it was noted that the pre‐stimulus phase of alpha oscillations can affect further processing during the activation of bottom‐up feature‐based attention prior to stimulus onset. Some authors suggested that the pre‐stimulus alpha phase modulated subsequent behavioral performance and that the preferred phase was different for high‐ versus low‐salience cues in alpha oscillations (Jia et al., 2022). However, the study involved a mixture effect of pre‐stimulus feature‐based attention and priming. Concerning the idea of sequential spatial sampling in attention, to our knowledge, evidence in favor of this mechanism in feature‐based attention is only available in post‐stimulus intervals (Mo et al., 2019). However, one must note that some findings about sequential spatial sampling in studies involving pre‐stimulus spatial attention could also be due to pre‐stimulus feature‐based attention due to the intermixed effects.

In addition to alpha, activation of beta oscillations prior to stimulus onset was reported in pre‐stimulus feature‐based attention, although not specifically in the motor cortex (Pagnotta et al., 2020). These authors found an increase in pre‐stimulus beta power in the prefrontal and occipito‐temporal regions. Furthermore, beta band synchronization in the regions involved in the processing of the upcoming stimulus in a pre‐stimulus feature‐based attention task was increased. They suggested that the observed pre‐stimulus changes in beta rhythms might control endogenous attentional mechanisms prior to the presentation of the target, ensuring that the relevant task information, like the cued feature, is transferred to downstream brain regions. Overall, while brain activity changes in pre‐stimulus feature‐based attention resemble initial findings of pre‐stimulus spatial attention, later debates expressed in pre‐stimulus spatial attention are rarely tested in pre‐stimulus feature‐based attention, and the examination of them deserves further investigations.

## 3 TEMPORAL ATTENTION

Attention is considered a dynamic process, modulated by prediction to prioritize and select pertinent information even before a stimulus appears (A. C. Nobre & Van Ede, 2018). Temporal structures—defined as “any repeating sets of intervals among two or more items”—that activate temporal attention are categorized into four types based on their creation: associations, hazard rates, rhythms, and sequences. All these structures can occur before stimulus onset, activating pre‐stimulus temporal attention. They often enhance other anticipated identifying attributes rather than operating in isolation (A. C. Nobre & Van Ede, 2018, 2023). Although these authors provided a detailed review, we will focus on the main pre‐stimulus oscillatory activities and the subsequent behavioral effects of these different forms of temporal attention.

### 3.1 Temporal association

Temporal association refers to predictive temporal relationships between upcoming stimuli and could be examined through symbolic temporal cues containing information about the likely occurrence time of an impending stimulus (A. C. Nobre & Rohenkohl, 2014; Los, 2010). In this paradigm, the interval before presenting a target, sometimes called the foreperiod, lasts about 600–1400 ms (K. Nobre, 2010; Niemi & Näätänen, 1981). Pre‐stimulus alpha power was attenuated across the visual areas when temporal cues informed participants about the timing of the incoming stimulus, particularly in shorter fore‐periods (Zanto et al., 2011). More recent work suggested that temporal cues enhance target identity representations and prevent interference caused by temporally adjacent distractor stimuli by providing a protective window for processing the target as well as attenuating posterior pre‐stimulus alpha power (Van Ede et al., 2018). However, the study found no evidence of anticipatory pre‐stimulus phase‐alignment and subsequent facilitation for target decoding. The attenuation of alpha power for valid versus neutral cues was consistent with the gating mechanism, where information about target onset facilitated further processing.

### 3.2 Hazard rate

Hazard rate refers to the conditional probability that an event will occur at a specific time before it actually happens (Luce, 1986). A common approach to assessing hazard rates in an experiment is to derive the occurrence time of the target from different temporal probability distributions across blocks of the experiment. When targets are presented with higher probability, participants’ expectations increase and vice versa. Higher pre‐stimulus temporal expectation leads to reduced reaction times (Andre M Cravo et al., 2011). This facilitation is preceded by an increase in pre‐stimulus theta power and coupling of theta phase with beta power before the target presentation. An fMRI study indicated that the left parietal cortex may play a key role in instantiating the behavioral benefits of hazard rates (Coull et al., 2016). These facilitations occurred even when participants were unaware of the time distribution, suggesting that an increase in theta power reflects enhanced anticipation and expectation, while the coupling of theta phase and beta power may represent pre‐stimulus motor preparation.

### 3.3 Rhythm

Rhythm refers to temporal regularities in an experiment. A typical example is a task design where stimuli are presented at regular versus irregular pre‐stimulus time intervals, enabling rhythmic temporal attention for regular, but not for irregular intervals (Breska & Deouell, 2014; Praamstra et al., 2006; Rohenkohl & Nobre, 2011). These authors reported attenuation of occipital alpha power for expected target times, particularly when short intervals preceded a visual stimulus, again compatible with the gating mechanism. They also showed that the phase of delta oscillations in the human visual cortex was predictive of the quality of target processing only in regular streams, where the optimal delta phase occurred in anticipation of expected events (André M Cravo et al., 2013; Schroeder & Lakatos, 2009). Delta‐phase entrainment was closely related to increased contrast gain, indicating that pre‐stimulus temporal rhythmic attention modulates perception through a gain mechanism and via contrast enhancement.

### 3.4 Sequence

Sequence refers to recurring events where the timing of the next anticipated stimulus depends on the position of the current stimulus in the sequence. Temporal sequences are often examined through modified versions of serial reaction time (SRT) tasks. In a classic SRT task, a sequence that includes a series of repeated targets/responses is represented multiple times (Nissen & Bullemer, 1987). However, a temporal sequence is created by adjusting the timing between stimuli in the sequence, typically without participants being aware of this adjustment. The sequence is then repeated multiple times in each session of the experiment. When utilized alone, temporal sequences are not found to enhance performance; however, when combined with an ordinal or spatial sequence, they lead to improvements in response time (O'Reilly et al., 2008; Shin & Ivry, 2002).

**FIGURE 4:**
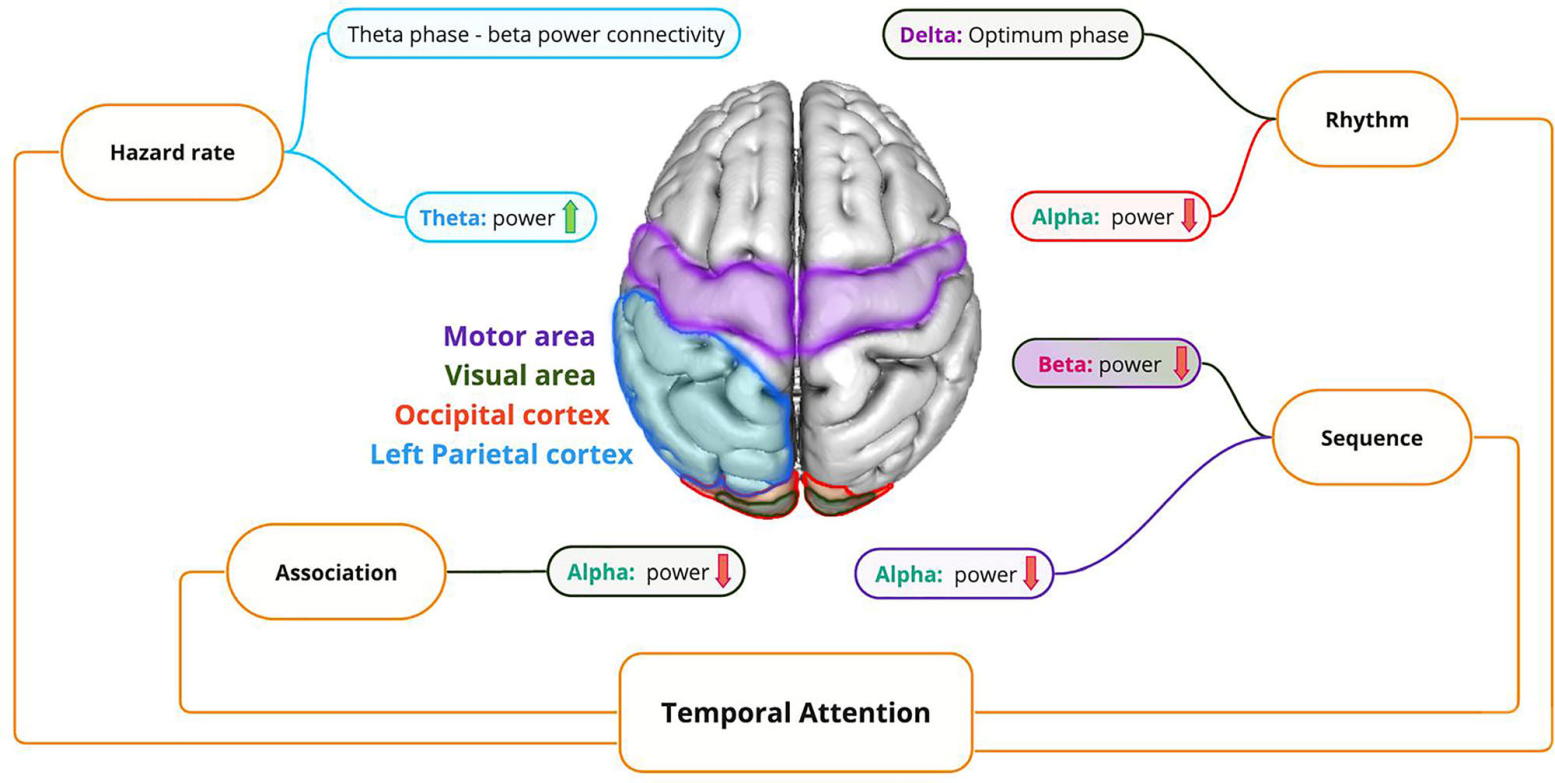
Concise presentation of oscillatory changes for various structures involved in pre‐stimulus temporal attention.

A more recent study examined the spatial and temporal anticipatory neural dynamics of sequences using a modified version of the SRT, where participants had to respond to different targets using specified fingers (S. G. Heideman et al., 2018). It was demonstrated that reaction times improved for repeated versus new sequences, with this improvement consistently larger for shorter intervals. The facilitation was preceded by the lateralization of motor cortical beta oscillations in visual and motor areas, manifesting as reduced beta power in contralateral versus ipsilateral regions in anticipation of the response associated with the forthcoming target's location and timing. This effect was more pronounced for repeated (learned) versus new (unlearned) sequences, as well as when comparing short versus long intervals. Additionally, pre‐stimulus alpha power was decreased in motor areas in anticipation of the target/motor timing. The reduction in alpha and beta power prior to stimulus onset aligns with the gating mechanism, which suggests that these oscillations prepare the brain for incoming stimuli by modulating sensory and motor processing. Figure 4 provides a schematic presentation of pre‐stimulus oscillatory activities in the context of pre‐stimulus temporal attention.

Interactions between different forms of temporal attention can occur, potentially amplifying or diminishing each other's effects (A. C. Nobre & van Ede, 2023). Notably, many studies focusing on pre‐stimulus conditions often overlook the role of temporal attention. For instance, numerous studies on pre‐stimulus spatial attention employ regular pre‐stimulus intervals, inadvertently engaging pre‐stimulus rhythmic temporal attention. Another common oversight is the varying probabilities of stimulus presentation without considering the impact of pre‐stimulus hazard rate temporal attention. Consequently, the outcomes reported in these studies may be influenced, at least in part, by pre‐stimulus temporal attention. While all experiments have limitations in controlling for every potential variable, it is crucial to recognize the intermingling effects of different cognitive phenomena that could affect pre‐stimulus oscillations. Furthermore, it remains unclear whether pre‐stimulus power changes are causally related to different forms of temporal attention, and whether similar or disparate effects are observed for distractor suppression compared to target facilitation across different forms of temporal attention.

## 4 AROUSAL

Arousal, also known as tonic alertness, vigilance, vigilant attention, or sustained attention, is defined as the general state of responsiveness to external stimuli and is typically considered when irregular onset asynchrony is present in tasks (Schroeder & Lakatos, 2009). Arousal is often measured by recording changes in pupil size or skin conductance (Lee et al., 2018).

The effect of pre‐stimulus arousal on stimulus processing is complex, partly because arousal is often not the sole cognitive function enabled prior to stimulus onset. For instance, in a study where pre‐stimulus cues were presented to the left or right of a fixation point, the discussion primarily focused on pre‐stimulus sustained attention, although pre‐stimulus covert spatial attention was also likely activated (Busch & VanRullen, 2010). Similarly, in a study employing regular time intervals between stimuli with a focus on arousal, pre‐stimulus temporal attention in a rhythmic structure would also have been activated (Mathewson et al., 2009). These examples suggest that it can be very difficult, if not impossible, to isolate the effects of pre‐stimulus arousal from other pre‐stimulus cognitive functions. Some studies have attempted to reduce this mixed effect by utilizing arousal‐specific objective measurements such as pupillometry or employing arousal cues prior to stimulus onset (Allen et al., 2016).

Conversely, multiple studies that did not directly attribute their findings to arousal still presented results that, when compared to similar task designs focusing on arousal, suggest that pre‐stimulus arousal was probably engaged. For instance, studies involving NT stimuli, where pre‐stimulus brain activity was predictive of conscious perception, or studies using ambiguous supra‐threshold stimuli, where perception fluctuated between two interpretations of the ambiguous stimulus across trials. In these contexts, changes in pre‐stimulus power, phase, or large‐scale synchrony were predictive of subsequent perception. For example, in a study where participants detected NT light pulses, lower pre‐stimulus alpha power in the relevant brain regions was associated with superior perceptual performance, compatible with the gating mechanism (Ergenoglu et al., 2004). Another study on the detection of NT contours surrounded by noise patterns found that the excitatory pre‐stimulus phase (and not the nonoptimal inhibitory pre‐stimulus phase) facilitated post‐stimulus information flow between higher and lower visual areas, leading to conscious perception (Hanslmayr et al., 2013). A more recent study demonstrated that pre‐stimulus phase‐based connectivity between lower and higher visual brain regions in alpha and beta frequency bands was predictive of subsequent perception in the Rubin face‐vase illusion (Rassi, Fuscà, et al., 2019). Similarly, an fMRI study using the same task revealed that an increased BOLD signal in the pre‐stimulus period in a face‐related brain region was associated with the perception of the face rather than the vase (Hesselmann et al., 2008).

We propose that innate temporal sampling is a crucial factor contributing to these predictions, facilitating participants’ perceptions to alternate between two outcomes, termed perceptual cycles (VanRullen, 2016). Another study utilizing a similar task design presented NT stimuli and recorded MEG signals and participants’ pupil size, yielding behavioral results consistent with those aforementioned studies based on perceptual cycles. Importantly, they used multivariate analysis of pre‐stimulus sensor‐level activities and identified a process correlated with changes in pupil size, thus attributed to arousal (Podvalny et al., 2019). Generalizing the findings of this latter study to those previously mentioned, we suggest that pre‐stimulus arousal could also be the primary cognitive function enabled in those studies, although this hypothesis requires further investigation to confirm.

## 5 MENTAL IMAGERY

Pre‐stimulus brain activities are sometimes linked to imagination. Multiple studies suggest that mental imagery operates similarly to weak perceptual stimuli, such as those with low contrast or luminance (Pearson, 2019). Although imagination is typically considered a voluntary process, it can also occur involuntarily (Pearson et al., 2015). Experiments that attribute pre‐stimulus activities to mental imagery can generally be divided into two main paradigms.

In the first paradigm, participants are instructed to engage in mental imagery before the presentation of a stimulus, with behavioral measurements assessing the effects of this pre‐stimulus imagination on stimulus processing (Chang & Pearson, 2018; Keogh & Pearson, 2018). A common approach involves instructing participants to perform mental imagery before a binocular rivalry task, where stimuli such as gratings with different directions or rotations are presented to each eye (Keogh & Pearson, 2018; Pearson et al., 2008, 2011). This methodology applies to both static (e.g., color and orientation) (Chang et al., 2013) and dynamic (e.g., motion imagery) (Chang & Pearson, 2018) visual features.

Using rivalry tasks to assess the impact of imagery offers several advantages. First, it allows for the evaluation of the sensory strength of mental imagery. Most research in this area relies on subjective reports like the questionnaire upon mental imagery (Betts, 1909; Sheehan, 1967) and the vividness of visual imagery questionnaire (Marks, 1973) to measure imagery strength. However, the influence of imagery on subsequent perception becomes clearer when objective measures are utilized. This can be achieved with an embedded probe task, where the probe in the dominant rivalry pattern is detected more easily than in the non‐dominant pattern, thus representing the influence of mental imagery on stimulus processing (Pearson, 2019). Second, a rivalry task design can differentiate perceptual bias from decisional bias. In the context of mental imagery, perceptual bias is defined as the proportion of trials in which the dominant stimulus matches the imagined stimulus. To distinguish this from non‐perceptual biases, catch trials can be included where no actual rival stimuli are presented to participants. If non‐perceptual factors, such as decisional bias due to pre‐stimulus cues, affect judgments, a statistically significant bias is expected in these catch trials as well (Chang & Pearson, 2018). Third, such a performance‐based dependent variable enables the measurement and comparison of the effects of different features like pure color or motion mental imagery (Chang & Pearson, 2018; Chang et al., 2013; Kwok et al., 2019).

In the second paradigm, individuals are not explicitly instructed to engage in mental imagery before the presentation of a stimulus. However, mental imagery is often considered a probable factor to explain behavioral differences arising from pre‐stimulus activities (Kok et al., 2014). Typically, these studies do not perform explicit measurements of imagination. Some researchers argue that the attribution of mental imagery to pre‐stimulus activities depends on how imagination is defined (Battistoni et al., 2017). If mental imagery is defined in a “broad sense”—as contemplating how something might look, rather than a clear and conscious imagination of an object—then it might be attributed to the pre‐stimulus effect. Additionally, it is important to consider the overlapping effects of pre‐stimulus mental imagery and pre‐stimulus feature‐based attention, as the concepts of sensory versus non‐sensory templates in feature‐based attention could be conflated with clear conscious imagination versus broad‐sense imagination. To this end, exploring the dissociative effects of mental imagery and attention on subsequent processing may be insightful. For example, extended mental imagery durations are associated with a stronger facilitatory effect on the perception of subsequent stimuli, whereas this is not observed with prolonged attention periods to a stimulus (Pearson et al., 2015). Moreover, imagination has a slower but more flexible facilitatory effect on the perception of an incoming stimulus compared to attention (Pearson et al., 2008).

Early studies indicated a facilitatory effect of pre‐stimulus imagery on reaction time and the discrimination threshold of a stimulus (Brochard et al., 2004; Ishai & Sagi, 1995). Additionally, pre‐stimulus mental imagery induces a priming effect on stimulus processing in both static and motion imagery binocular rivalry designs, with the duration of imagery correlating with the strength of the priming effect on stimulus perception (Pearson, 2014). This effect is location‐dependent for stationary imagery rivalry, but it is not confined to the imagined location for motion mental imaging rivalry (Chang & Pearson, 2018).

The spectrum of imagery, ranging from weak to highly vivid imagination—aphantasia versus hyperphantasia (Galton, 1880)—can activate pre‐stimulus mental imagery in many task designs. Therefore, researchers may need to implement specific controls to manage mental imagery when designing pre‐stimulus tasks. Furthermore, to our knowledge, no electrophysiology study has yet specifically tested pre‐stimulus mental imagery, leaving the role of different frequency bands in pre‐stimulus imagination as an open question. Employing high temporal resolution recording techniques such as M/EEG is crucial in this field. Additionally, techniques such as repetitive transcranial magnetic stimulation or transcranial alternating current stimulation could further enhance control over various types of imagination prior to stimulus onset.

## 6 EXPECTATIONS

In the context of pre‐stimulus brain activities, several studies have explored how expectation and attention are concurrently activated before stimulus onset. To differentiate these concepts, it is suggested that expectation should be defined as the probability of a stimulus occurrence, while attention relates to task relevance (C. Summerfield & Egner, 2009). Although it is possible to separate the effects of expectation and attention (Marzecová et al., 2017; Tal‐Perry & Yuval‐Greenberg, 2021), they are often tightly coupled and exert a modulatory influence on each other. Expectation has also been described as “dynamic content‐specific information about a previously experienced sensory environment” (Podvalny et al., 2019). Beyond past experiences, factors such as experimental context and genetics may influence how expectations are formed (Bar, 2009; C. Summerfield & De Lange, 2014). Typically, expectation is seen as a top‐down phenomenon, but bottom‐up facilitation, such as the priming effect in block paradigms, may also occur (Noonan et al., 2016).

Focusing on pre‐stimulus brain activities, any cognitive function enabled prior to stimulus onset can consciously or unconsciously modulate participants' expectations. This modulation can occur through various techniques discussed in this review, including altering the probability of stimulus presentation over trials (Marzecová et al., 2017), presenting a cue containing information about the upcoming stimulus (A. C. Nobre & Rohenkohl, 2014; Noonan et al., 2016), asking subjects to imagine a stimulus before its actual presentation (Chang & Pearson, 2018; Keogh & Pearson, 2018), using regular stimulus onset asynchrony (S. Heideman, 2017), and providing an association between a forthcoming stimulus and an already presented stimulus (Meijs et al., 2018, 2019). In these scenarios, the expectation can be valid, correctly guiding the participant to the incoming stimulus, or invalid, creating a state of violated expectation.

This study explores various changes that can occur prior to stimulus onset in response to this modulation, such as alterations in BOLD response, phase adjustments (Ronconi & Marotti, 2017), power increases or decreases (Benwell et al., 2017; Foster & Awh, 2019), changes in functional connectivity (Pagnotta et al., 2020; Rassi, Wutz, et al., 2019), and changes in pupil size (Murphy et al., 2014). A variety of techniques may be used to test the existence and role of different kinds of expectations, from simple statistical tests to more complex models (Allen et al., 2016; Rassi, Fuscà, et al., 2019) or machine learning methods (Railo et al., 2020).

Multiple important considerations arise when focusing on pre‐stimulus expectation. First, the effects of expectation on later processing are highly dependent on task design and its purpose. For example, in tasks involving learning, anticipated events may be seen as redundant, leading to a decrease in processing demand—a topic mainly discussed within the framework of predictive coding, which is beyond the scope of this review. Additionally, depending on the task design, the effect of expectation on perception may manifest at different levels of the cortical hierarchy, such as sensory, semantic, or decision‐making layers. Finally, how to measure the effect of expectation is also crucial and depends on the hypothesis being tested. For instance, scalp brain recordings may not detect the influence of expectation on activity formed in deep brain structures.

## 7 PRE‐STIMULUS CUES AND TIME INTERVAL

Selecting a cue before a stimulus to modulate a cognitive function requires careful consideration. Additionally, how to consider the pre‐stimulus time interval is also of utmost importance. Besides what we have already discussed, paying attention to the following points can facilitate a clearer interpretation of results:

1. *Cue presentation and priming effects*: The type of cue can trigger different degrees of priming. For instance, using exact image cues (e.g., a red circle to indicate the color of the forthcoming stimulus) has been shown to result in pure priming effects in many studies (Theeuwes, 2013). To minimize such effects, symbolic cues (e.g., a word expressing a feature or location) are recommended, although they do not completely eliminate priming. This is because semantic representations can facilitate perceptual processing of the primed object through sensory systems (Chen & Spence, 2011). Priming is also prevalent in block designs and inter‐trial setups, where avoiding the presentation of the same stimulus in consecutive trials can help mitigate inter‐trial priming (Lamy & Kristjánsson, 2013; Theeuwes & Van der Burg, 2011).
2. *Automated symbolic attention orienting*: Overlearned symbolic cues like arrows can induce automated attention orienting, even if they carry no task‐relevant information (Ristic & Kingstone, 2012). Cues that have been informative in the past tend to be more rapidly learned and more influential (Griffiths & Le Pelley, 2019).
3. *Pre‐stimulus time interval selection*: The choice of pre‐stimulus time intervals varies significantly across studies, from a few hundred milliseconds in electrophysiology studies to several seconds in fMRI studies. While most studies continue the pre‐stimulus interval up to stimulus onset, some end it a few seconds or milliseconds before the stimulus appears (Rouhinen et al., 2020). In attention paradigms, typical intervals are around 100 ms for exogenous and about 500 ms for endogenous attention (Giordano et al., 2009; Nakayama & Mackeben, 1989). However, specific definitions for other cognitive functions like feature‐based attention, arousal, and mental imagery are less established. More pre‐registered studies are needed to standardize pre‐stimulus interval selection and provide appropriate criteria for different contexts (Ruzzoli et al., 2019).
4. *Time‐frequency analysis considerations*: Caution is necessary when performing time‐frequency analysis if the pre‐stimulus interval is too close to the stimulus onset. Time‐frequency filtering involves some degree of frequency and temporal smoothing, which can cause changes in post‐stimulus power or phase to leak into the pre‐stimulus interval. This may result in misattributing early post‐stimulus activities as pre‐stimulus effects.

These points highlight the need for precise methodological decisions to avoid confounding factors and for accurate interpretation of pre‐stimulus brain activities.

## 8 CONCLUSION

**FIGURE 5:**
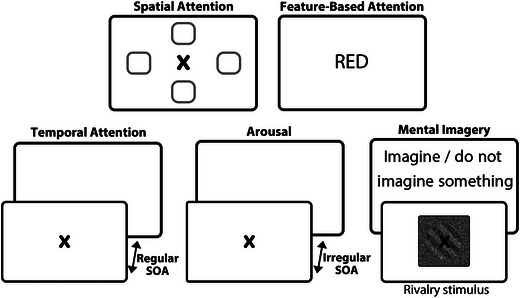
Schematic representation of the main characteristic for different cognitive functions enabled in the pre‐stimulus interval. Only rhythmic temporal attention is shown in this figure to dissociate it from arousal. SOA, stimulus onset asynchrony.

The findings presented in this review highlight a range of cognitive phenomena that may be activated during the pre‐stimulus interval, including spatial and feature‐based attention, temporal attention, arousal, and mental imagery. Figure 5 illustrates the main dissociative characteristics of these cognitive functions, providing a visual representation that enhances understanding of their distinct and overlapping effects.

We propose that the specific pre‐stimulus cognitive function activated and the exact role of pre‐stimulus brain activities in these cognitive functions are highly contingent upon the task design and the objectives of the experiment. It is crucial for researchers to recognize that multiple cognitive processes are typically involved in the pre‐stimulus period. Thus, experimental designs must be meticulously crafted to control confounding factors, enabling the clear disentanglement of different cognitive processes. Our findings suggest that trial‐to‐trial perceptual variability is often attributable to variations in the power, phase, or connectivity of pre‐stimulus oscillations, in the context of the enabled pre‐stimulus cognitive function. Although common mechanisms have been frequently reported to account for the effects of changes in pre‐stimulus brain activities on subsequent processing with regard to the activated pre‐stimulus cognitive function, our review indicates that no single mechanism can fully explain all observed effects. Instead, it appears that multiple mechanisms may operate concurrently to facilitate effective interactions between pre‐stimulus brain activities and subsequent cognitive processing.

This review is limited in its scope. First, it is confined to human studies within the visual domain, although significant research on pre‐stimulus brain activities also exists in other modalities and in non‐human primates. Second, while focusing on cognitive functions activated in the pre‐stimulus period and their subsequent impact on behavioral performance, we did not explore how post‐stimulus cognitive functions might be influenced by earlier brain activities. Third, this study does not address the role of working memory, another critical pre‐stimulus cognitive function.

Ultimately, our understanding of pre‐stimulus cognitive functions and the associated changes in pre‐stimulus brain activities remains fragmented and incomplete. Many questions linger, necessitating systematic studies with well‐designed tasks to forge a more comprehensive understanding of how pre‐stimulus oscillations influence post‐stimulus processing in relation to the cognitive functions activated prior to stimulus onset. To advance this field, we propose the following questions for future research:

1. Distractor suppression versus target facilitation: Is distractor suppression fundamentally different from target facilitation in pre‐stimulus feature‐based attention?
2. Beta frequency band in motor cortex: Does the beta frequency band change in motor cortices when pre‐stimulus feature‐based attention is enabled?
3. Alpha phase in pre‐stimulus feature‐based attention: When separating pre‐stimulus feature‐based attention from priming, spatial, and temporal attention, does the pre‐stimulus phase of alpha oscillations modulate subsequent processing?
4. Sequential sampling in feature‐based attention: Is sequential sampling absent in pre‐stimulus feature‐based attention?
5. Causal role of alpha power on feature‐based attention: Is there any causal relationship between changes in pre‐stimulus alpha power and pre‐stimulus feature‐based attention?
6. Facilitation and suppression in temporal attention: Are the effects of target facilitation different from distractor suppression in various forms of temporal attention?
7. Causality in temporal attention: Are the observed changes in pre‐stimulus oscillatory activities causally related to temporal attention, or are they a by‐product?
8. Arousal and mechanisms: How is pre‐stimulus arousal related to the mechanisms reviewed in this article?
9. Imagination and pre‐stimulus oscillations: How do pre‐stimulus oscillatory activities change when participants are asked to imagine something prior to stimulus onset?
10. Overlap with resting‐state activities: What about the overlap and discrepancies between pre‐stimulus and resting‐state brain activities? Is there any specific pre‐stimulus brain network akin to those observed in resting‐state networks?
11. Clinical and practical applications: What are the clinical implications of pre‐stimulus brain activities? How might they play a role in applications such as neurofeedback or neuromarketing?
12. Criticality of the pre‐stimulus interval: Is the pre‐stimulus period always critical for stimulus processing, or are there exceptions?

These questions are designed to not only probe the existing boundaries of knowledge but also to encourage methodological innovations and interdisciplinary approaches. This structured approach aids in highlighting the multifaceted nature of pre‐stimulus brain activities and their potential impacts across different cognitive and clinical domains.

## AUTHOR CONTRIBUTIONS

**Narjes Soltani Dehaghani**: Writing—original draft; conceptualization; investigation; visualization. **Mojtaba Zarei**: Conceptualization; methodology; supervision; visualization; writing—review and editing; investigation; funding acquisition.

### PEER REVIEW

The peer review history for this article is available at https://publons.com/publon/10.1002/brb3.3654.

## Data Availability

Data in support of any claim in this review are found in the relevant citation.
